# Draft Genome Sequence of *Methylobacterium mesophilicum* Strain SR1.6/6, Isolated from *Citrus sinensis*

**DOI:** 10.1128/genomeA.00356-13

**Published:** 2013-06-20

**Authors:** Diogo Marinho Almeida, Francisco Dini-Andreote, Aline Aparecida Camargo Neves, Rommel Thiago Jucá Ramos, Fernando Dini Andreote, Adriana Ribeiro Carneiro, André Oliveira de Souza Lima, Pablo Henrique Caracciolo Gomes de Sá, Maria Silvanira Ribeiro Barbosa, Welington Luiz Araújo, Artur Silva

**Affiliations:** Instituto de Ciências Biológicas, Universidade Federal do Pará, Belém, Pará, Brazila; Departamento de Microbiologia, Instituto de Ciências Biomédicas, Universidade de São Paulo, Biomédicas II, Cidade Universitária, São Paulo, São Paulo, Brazilb; Departamento de Genética, Escola Superior de Agricultura Luiz de Queiroz, Universidade de São Paulo, Piracicaba, São Paulo, Brazilc; Departamento de Solo, Escola Superior de Agricultura Luiz de Queiroz, Universidade de São Paulo, Piracicaba, São Paulo, Brazild; Centro de Ciências Tecnológicas da Terra e do Mar, Universidade do Vale do Itajaí, Itajaí-Santa Catarina, Brazile

## Abstract

*Methylobacterium mesophilicum* strain SR1.6/6 is an endophytic bacterium isolated from a surface-sterilized *Citrus sinensis* branch. Ecological and biotechnological aspects of this bacterium, such as the genes involved in its association with the host plant and the primary oxidation of methanol, were annotated in the draft genome.

## GENOME ANNOUNCEMENT

The pink-pigmented facultative methylotrophic bacterium *Methylobacterium mesophilicum* (*Alphaproteobacteria*) is ubiquitous on the surface and the interior of plants (1, 2). Members of this genus are able to grow on one or several reduced one-carbon compounds (C_1_), such as methylamine and methanol, which is a volatile organic compound produced by plants, and also induce plant growth (3). *M. mesophilicum* strain SR1.6/6 was isolated from *Citrus sinensis*, and an interaction with *Xylella fastidiosa*, the causal agent of CVC (citrus-variegated chlorosis), has been proposed (1, 4, 5). Also, *M. mesophilicum* SR1.6/6 produces at least six long-chain acyl-homoserine lactones (HSLs), and can be transmitted by *Bucephalogonia xanthophis*, a sharpshooter insect (6, 7), suggesting that this strain is able to interact with different host and microbial species.

Genome sequencing was carried out by Genome Sequencer FLX 454 Titanium/Roche, resulting in a total of 253,143,785 bp, and analyzed 635,612 reads, with a coverage of 40×. The closest related available genome of *Methylobacterium radiotolerans* strain JCM2831 was used as a reference during the assembly process. The adapters and low-quality sequences were removed using the scripts “sff_extract_0_2_13.py” (http://bioinf.comav.upv.es/sff_extract/), for generation of the “traceinfo.xml,” and filtered with the script “pyseqfilter.py,” considering reads with 20 in Phred quality. We also performed *de novo* assembly using Velvet (8) and MIRA (9), generating a total of 1,015 contigs with N_50_ of 38,663 bp.

The scaffolds were obtained by curing and extending the contigs with the SeqMan NGen and SeqMan software programs (DNASTAR). The nonextended contigs were mapped using the CLC Genomics Workbench (CLC bio, Inc.) for *de novo* assembly and further extension. This recursive approach (totaling 3 interactions) allowed the generation of 29 scaffolds with a total length of 6,214,449 bp, with an *N*_50_ of 333,386.

The functional annotation was performed using FgenesB (SoftBerry), RNAmmer (10), tRNAscan-SE (11), Tandem Repeats Finder (http://tandem.bu.edu/trf/trf.html), and InterProScan (12). In addition, manual annotation was also performed using Artemis software (13).

This genome of this bacterium was found to be 6,214,449 bp long, containing 5,945 putative open reading frames. The average G+C content is 69.47% with 46 tRNAs, 4 rRNA genes, and 57 pseudogenes. We annotated gene clusters that enable methanol oxidation, which is a known metabolic process that is activated during plant-methylotrophic bacterium interaction (14). Interestingly, we found that the *pqqA* gene, which is not essential for C_1_ growth, is missing, and *mxaF*, which is essential for C_1_ growth, is duplicated. Other genes are located in 4 clusters on the chromosome with a similar organization observed for *Methylobacterium extorquens* AM1 (15). Cluster 1 (2.8 kb) contains the genes *mxcQE* that codify a sensor kinase and a two-component LuxR family transcriptional regulator, respectively. In cluster 2 (3.488 kb), the genes *pqqBC* and *pqqDE* are located 344 bp downstream of the gene *mxbDM* that is related to the transcriptional regulation of methanol dehydrogenase (MeDH) (15). Cluster 3 is a small region (2.808 kb) that contains the *pqqFG* gene (PQQ biosynthesis), which has been described to occur in a separated metabolic module (15). Cluster 4 (11.718 kb) is transcribed in the opposite direction of the other genes. This cluster contains 14 genes (*mxaFJGIRSACKLDEHB*) that are responsible for codifying the structural polypeptides of methanol dehydrogenase (15).

### Nucleotide sequence accession numbers.

The *M. mesophilicum* strain SR1.6/6 genome sequence and annotation data have been deposited at DDBJ/EMBL/GenBank under the accession no. ANPA00000000. The version described in this paper is the first version, accession no. ANPA01000000.
